# A Comparative Study of Phase I and II Hepatic Microsomal Biotransformation of Phenol in Three Species of Salmonidae: Hydroquinone, Catechol, and Phenylglucuronide Formation

**DOI:** 10.3390/fishes9070284

**Published:** 2024-07-17

**Authors:** Richard C. Kolanczyk, Laura E. Solem, Patricia K. Schmieder, James M. McKim

**Affiliations:** 1 United States Environmental Protection Agency, Office of Research and Development, Center for Computational Toxicology and Exposure, Great Lakes Toxicology and Ecology Division, 6201 Congdon Boulevard, Duluth, MN 55804, USA; 2 National Research Council, 6201 Congdon Boulevard, Duluth, MN 55804, USA

**Keywords:** phenol, metabolic activation, metabolic deactivation, microsomes, Km and Vmax, fish, rainbow trout, lake trout, brook trout

## Abstract

The *in vitro* biotransformation of phenol at 11 °C was studied using pre-spawn adult rainbow (*Oncorhynchus mykiss*) (RBT), brook (*Salvelinus fontinalis*) (BKT), and lake trout (*Salvelinus namaycush*) (LKT) hepatic microsomal preparations. The incubations were optimized for time, cofactor concentration, pH, and microsomal protein concentration. Formation of Phase I ring-hydroxylation and Phase II glucuronidation metabolites was quantified using HPLC with dual-channel electrochemical and UV detection. The biotransformation of phenol over a range of substrate concentrations (1 to 180 mM) was quantified, and the Michaelis–Menten kinetics constants, Km and Vmax, for the formation of hydroquinone (HQ), catechol (CAT), and phenylglucuronide (PG) were calculated. Species differences were noted in the Km values for Phase I enzyme production of HQ and CAT, with the following rank order of apparent enzyme affinity for substrate: RBT > BKT = LKT. However, no apparent differences in the Km for Phase II metabolism of phenol to PG were detected. Conversely, while there were no apparent differences in Vmax between species for HQ or CAT formation, the apparent maximum capacity for PG formation was significantly less in LKT than that observed for RBT and BKT. These experiments provide a means to quantify metabolic activation and deactivation of xenobiotics in fish, to compare activation and deactivation reactions across species, and to act as a guide for future predictions of new chemical biotransformation pathways and rates in fish. These experiments provided the necessary rate and capacity (Km and Vmax) inputs that are required to parameterize a fish physiologically based toxicokinetic (PB-TK) model for a reactive chemical that is readily biotransformed, such as phenol. In the future, an extensive database of these rate and capacity parameters on important fish species for selected chemical structures will be needed to allow the effective use of predictive models for reactive, biotransformation chemicals in aquatic toxicology and environmental risk assessment.

## 1. Introduction

The identification and quantification of the biotransformation products of exogenous chemicals in aquatic animals is needed to advance environmental risk assessments for bioaccumulative and reactive chemicals [1]. To accomplish this requires the selection of *in vitro/in vivo* methods that can provide information on necessary biotransformation pathway(s), rate, and capacity. Fish PB-TK models that address chemicals that are not readily biotransformed have been successfully developed for several fish species and are available for use in environmental risk assessment [2–9]. Model development has incorporated hepatic biotransformation by either assuming a linear first-order rate that is proportional to hepatic clearance or as saturable Michaelis–Menten kinetic Vmax and Km values [10–17]. The rate constants are for a single metabolite confined to the liver, which is considered the primary site for biotransformation. A survey of the literature for Michaelis–Menten rate constants by Fitzsimmons and co-workers [18] resulted in a compilation of the available *in vitro* rate and affinity values for xenobiotic metabolism in fish. The available Km and Vmax values were sorted with respect to species and chemical. The survey of limited values demonstrated a need for additional data. A limiting issue for the PB-TK modeling of readily metabolized chemicals is the lack of species-specific fish hepatic rate and capacity parameters (Km and Vmax) for the production of metabolites. Due to the lack of species-specific data for a given chemical biotransformation, surrogate fish and mammalian rate constants have been used as parameterization values in PB-TK models [13,16]. The use of species-specific values to achieve the most accurate model predictions is important, especially if the modeled metabolite results in the formation of a chemical species more reactive and toxic than the parent chemical.

One aspect of species extrapolation for assessing ecological risk that can be relevant both to exposure and effect characterizations is understanding xenobiotic metabolism, both as a means of detoxification as well as bioactivation. The role of metabolism in chemical detoxification and elimination is important, as it influences chemical bioaccumulation. Metabolism is generally defined as Phase I (bioactivation) and Phase II (detoxification) reactions as mediated by specific enzymes. Phase I metabolism reaction types are usually associated with reduction, oxidation, dealkylation, and hydrolysis of the parent chemical, while Phase II reactions typically include the formation of sulfate or glucuronide conjugates. The cytochrome P450 (Phase I) and glucuronosyltransferase (Phase II) enzymes are found in the liver microsomal fraction.

Species comparative Phase I biotransformation studies on benzo(a)pyrene (B(a)P) with the brown bullhead, black bullhead, mirror carp, trout, and channel catfish microsomes resulted in qualitatively similar metabolites by all species; however, the quantity of each metabolite formed was quite different across species [19–21]. The actual rates of biotransformation cited by the authors were difficult to compare or use in modeling, as no standardized rate and capacity parameters (Km and Vmax) were given. Most of the rates were single values determined at one substrate concentration. In many cases, differences in experimental procedures (i.e., physiological temperature) and microsomal assay methods also made it difficult to compare their results across species.

As in mammals, glucuronidation represents a major Phase II pathway of conjugative biotransformation in fish [22]. Generally, the potency of most metabolically activated xenobiotics is dependent on balancing the production of activated metabolites, which are usually catalyzed by Phase I (oxidative) enzymes, and the elimination of conjugated forms as catalyzed by Phase II enzymes. For example, the selective toxicity of 3-trifluoromethyl-4-nitrophenol (TFM) to sea lamprey was demonstrated and subsequently explained by a reduced capacity to glucuronidate and eliminate the lampricide in this species as compared with rainbow trout [23].

Most fish microsomal studies characterizing UDP-glucuronosyltransferase activity have used 4-nitrophenol as a standard substrate due to the ease of the assay [24–26]. Glucuronidation of other phenolic substrates using fish microsomes has been reported, such as TFM [27,28], 1-naphthol [24,25,29], and phenolphthalein [25]. But generally, these comparisons were measured for a single concentration of substrate, and no rate and capacity parameters (Km and Vmax) were provided that could be used in predictive PB-TK fish models. Interspecies comparisons of glucuronosyltransferase activity were also made difficult, as the assay conditions and temperatures used by different laboratories varied considerably. A significant amount of comparative Phase II data was collected for fish glucuronidation of 4-nitrophenol across a wide variety of species [30–34], but again, the rapid and easy-to-perform colorimetric assay for 4-nitrophenylglucuronide formation was typically measured at 25 °C, regardless of the fishes’ physiological temperature, using only one substrate concentration. Thus, if an accurate and reliable future Phase I and II biotransformation database of Km and Vmax values for selected chemicals is to be developed for extrapolating the toxic chemical potential among species, problems with varying methodologies and experimental protocols must be overcome.

Phenolic compounds as found in surface waters are considered a main environmental contaminant based upon the volume detected and the impact on providing safe drinking water, as well as potential fish toxicity [35,36]. Specifically, phenol was selected for these studies because (1) the acute and chronic toxicities were well known for fish [37,38], (2) the basic Phase I and II hepatic biotransformation schemes were in place for several fish species [39–44], and (3) a technique was developed for the sensitive detection of reactive metabolites (i.e., HQ and benzoquinone) at low rates of formation observed in rainbow trout microsomes [45].

The selection of three relatively close cold water fish species: rainbow, brook, and lake trout provide insight into species comparison across the Salmonidae family for the potential extrapolation of rate constants where the data are lacking. The rate and capacity parameters for phenol Phase I (ring-hydroxylation) and Phase II (glucuronidation) biotransformation were calculated for future incorporation into fish PB-TK modeling of phenol. All phenol experiments were performed on adult fish of similar size and age; held three months under the same food, water, and physiological temperature regimes; and tested by the same investigators using identical *in vitro* microsomal assays. Only through comparative data collected in this manner will an accurate evaluation between important species be attainable and allow subtle but important differences to be detected. The presented research allowed comparisons of potential metabolic activation and deactivation across three salmonid species by identifying the primary Phase I and II metabolites, quantified rates of formation for use in species extrapolation, and furthered our understanding of bioactivation as a component of species susceptibility to reactive chemicals.

## 2. Methods

### 2.1. Chemicals

Phenol, hydroquinone (HQ), benzoquinone (BQ), and catechol (CAT) were obtained from Aldrich Chemical Company (Milwaukee, WI, USA). Phenylglucuronide (PG), reducing equivalents, magnesium chloride (MgCl_2_), uridine 5′-diphosphoglucuronic acid (UDPGA), buffer components, G-6-P dehydrogenase, and 7-ethoxyresorufin were purchased from Sigma Chemical Co. (St. Louis, MO, USA). Acetonitrile and methanol from Burdick and Jackson (Muskegon, MI, USA) were of analytical grade. Resorufin was obtained from Pierce Chemical Company (Rockford, IL, USA). Disodium ethylenediaminete-traacetate and sodium dithionite were purchased from Fisher Scientific (Eden Prairie, MN, USA).

### 2.2. Standard and Sample Preparation and Handling

Standards and samples required special precautions to ensure stability. In all cases, cold solvents, buffers, and water were used to solubilize solid compounds. Solutions were kept on ice and protected from the light to avoid degradation of the analytes [45]. HQ, CAT, and BQ standard solutions were prepared daily in acetonitrile:water (1:1).

### 2.3. Animals

Adult rainbow trout (RBT) (500–1000 g) from Seven Pines Fish Hatchery (Lewis, WI, USA), brook trout (BKT) (750–1500 g) from Stockton Hatchery (Winona, MN, USA), and lake trout (LKT) (500–1000 g) from the Minnesota DNR Hatchery (Peterson, MN, USA) were fed commercial trout chow from Nelson and Sons Inc., (Murray, UT, USA) and held at 11 °C in flowthrough 815 L tanks with sand-filtered Lake Superior water (4 L/min). The adult fish, age over two years old, were in the pre-spawn state with respect to their sexual maturity. It was critical to the success of these experiments that the three species were held a minimum of three months on the same food and in the same water to avoid the possibility of differential induction of P450 in any of the species. To further guard against environmental enzyme induction, all fish were obtained from hatcheries utilizing ground water springs as their water source.

### 2.4. Microsomal Characterization

Liver microsomes were prepared from 24-h fasted trout. Livers from individual trout were homogenized for the preparation of each of six microsomal preparations per species of trout [45]. Isolated microsomes were stored at −80 °C for up to six months [46]. Each microsomal preparation was characterized as to the total protein [47], P450 content, and microsomal protein as measured by Estabrook et al. [48] using an extinction coefficient of 0.1 mM^−1^cm^−1^. The modified method of Pohl and Fouts [49] was used to determine 7-ethoxyresorufin-O-deethylase (EROD) activity utilizing excitation and emission wavelengths of 530 and 585 nm, respectively. The EROD reaction product that formed after 10 min at 11 °C was quantified against a resorufin standard curve.

### 2.5. Phase I Microsomal Incubations

The assay conditions for Phase I oxidation reactions were optimized for pH, incubation time, cofactor, and microsomal protein concentrations. Optimum HQ formation occurred at pH 8.0 for RBT and BKT and pH 8.5 for LKT. Catechol formation was favored at pH 8.0 for all three species, corresponding to the plasma pH for trout. The rates of HQ and CAT production with the concentrations of cofactors utilized were found to be linear with respect to time up to 20 min. No HQ or CAT formation was detected when phenol or one or more cofactors were removed from the incubation mixture, thus verifying absence of endogenous production of compounds that could interfere with detection of metabolites of interest.

Incubations (0.5 mL total volume) of each of nine phenol substrate concentrations (1 to 250 mM) with triplicate determinations over six individual fish microsomal preparations for each species were conducted in open microcentrifuge tubes at 11 °C with MgCl_2_ (20 mM), glucose-6-phosphate (10 mM), NADP (13.5 mM), G-6-P dehydrogenase (10 units), and microsomes (0.75 mg/mL protein) in 0.1 M Trizma-HCl buffer (pH 8.0) for 15 min. Microsomes and cofactors were incubated for 5 min prior to initiation of the reaction by addition of substrate. At completion, 0.05 mL cold Ba(OH)_2_ (saturated) and 0.05 mL cold ZnSO_4_ (25%) were added to the incubate to precipitate proteins. The samples were then vortexed, stored on ice for 5 min, and centrifuged for 3 min at 18,200× *g*. Samples were placed back on ice for 5 min and centrifuged 3 min at 18,200× *g*. Supernatant was transferred to amber HPLC vials equipped with inserts, maintained at 4 °C, and analyzed immediately by HPLC to preserve sample integrity.

### 2.6. Phase II Microsomal Incubations

The microsomal Phase II assay conditions were also optimized for pH, incubation time, cofactor, and microsomal protein concentration. Optimum PG formation occurred over a range of pH 7.5 to 8.0 for all three species: RBT, BKT, and LKT. A pH of 8.0 was used, consistent with the plasma pH for trout and Phase I assay conditions. The rate of PG production was found to be linear with respect to time up to 35 min with the concentration of cofactors utilized. No glucuronide formation was detected in the absence of phenol or cofactors.

Incubations (0.5 mL total volume) of each of nine phenol substrate concentrations (1 to 60 mM) with triplicate determinations over six individual fish microsomal preparations for each species were conducted in open microcentrifuge tubes at 11 °C with MgCl_2_ (20 mM), UDPGA (8 mM), and microsomes (0.75 mg/mL protein) in 0.1 M Trizma-HCl buffer (pH 8.0) for 20 min. The reaction was initiated by the addition of UDPGA. At completion of the reaction, samples were processed for HPLC analysis as detailed in the section above (Section 2.5. Phase I Microsomal Incubations).

### 2.7. Metabolite Identification and Quantification

The formation of HQ and CAT was quantified as described by Kolanczyk and Schmieder [44], using HPLC separation with electrochemical detection. Analysis of microsomal incubation samples and standards for PG was performed by HPLC as previously described for HQ and CAT but with UV detection at 265 nm. Electrochemical detector stability and instrument performance were assessed daily. The measurement of HQ, CAT, and PG in microsomal samples was based on the respective standard curves.

### 2.8. Data Analysis

For most experiments, data were expressed as the mean ± standard error of triplicate observations. The apparent kinetic parameters (Km and Vmax ± standard error) describing ring-hydroxylation and glucuronidation of phenol were determined based on direct measurement of metabolite formation (HQ, CAT, and PG). Michaelis–Menten rate constants, Km and Vmax, were calculated based on the inclusion of sufficient data points to define saturation. If data points are selectively eliminated from the rate calculation based solely on an assumption of enzyme inhibition, the estimates of Km and Vmax become artificially high without sufficient data to establish a plateau. The inclusion of multiple observations of declining rate may result in an underestimation and pull the curve too low, perhaps below the maximum rate of production at a given phenol concentration. Therefore, sufficient data points were included to adequately define the plateau of the curve and provide rate constants that are consistent with the measured data with respect to a maximum velocity. Parameter estimation (Km and Vmax) was done on data sets generated from individual fish, as well as a pooled data generated by averaging across six fish per species. No large differences were noted between these two methods of parameter estimation; therefore, all Km and Vmax values were fitted to the combined average rate of the six microsomal preparations from individual trout at each phenol concentration tested. A non-linear least squares regression program (EZ-Fit^™^ version 5.03; Perrella Scientific, Amherst, NH, USA) was used to fit untransformed kinetic data. Statistical comparisons between groups (*n* = 6) were performed using the unpaired *t*-test or one-way ANOVA at *p* ≤ 0.05.

## 3. Results

### 3.1. Microsome Characterization and Assay Optimization

Microsomes isolated from the livers of RBT, BKT, and LKT were characterized for microsomal and P450 protein content and for EROD activity. Rainbow trout had a significantly higher microsomal protein content (17.0 ± 0.9 mg/g liver) when compared to either BKT (9.1 ± 0.5 mg/g liver) or LKT 10.8 ± 0.8 mg/g liver) (Table 1). Male RBT and male BKT had significantly higher P450 protein content than females of the same species and some of the highest EROD activities, although considerable variations between individual fish were noted. No significant (*p* ≤ 0.05) differences in P450 protein levels (nmoles/mg microsomal protein) or EROD activity (pmoles/min/mg protein) were detected between species (Table 1).

**Figure 1.**
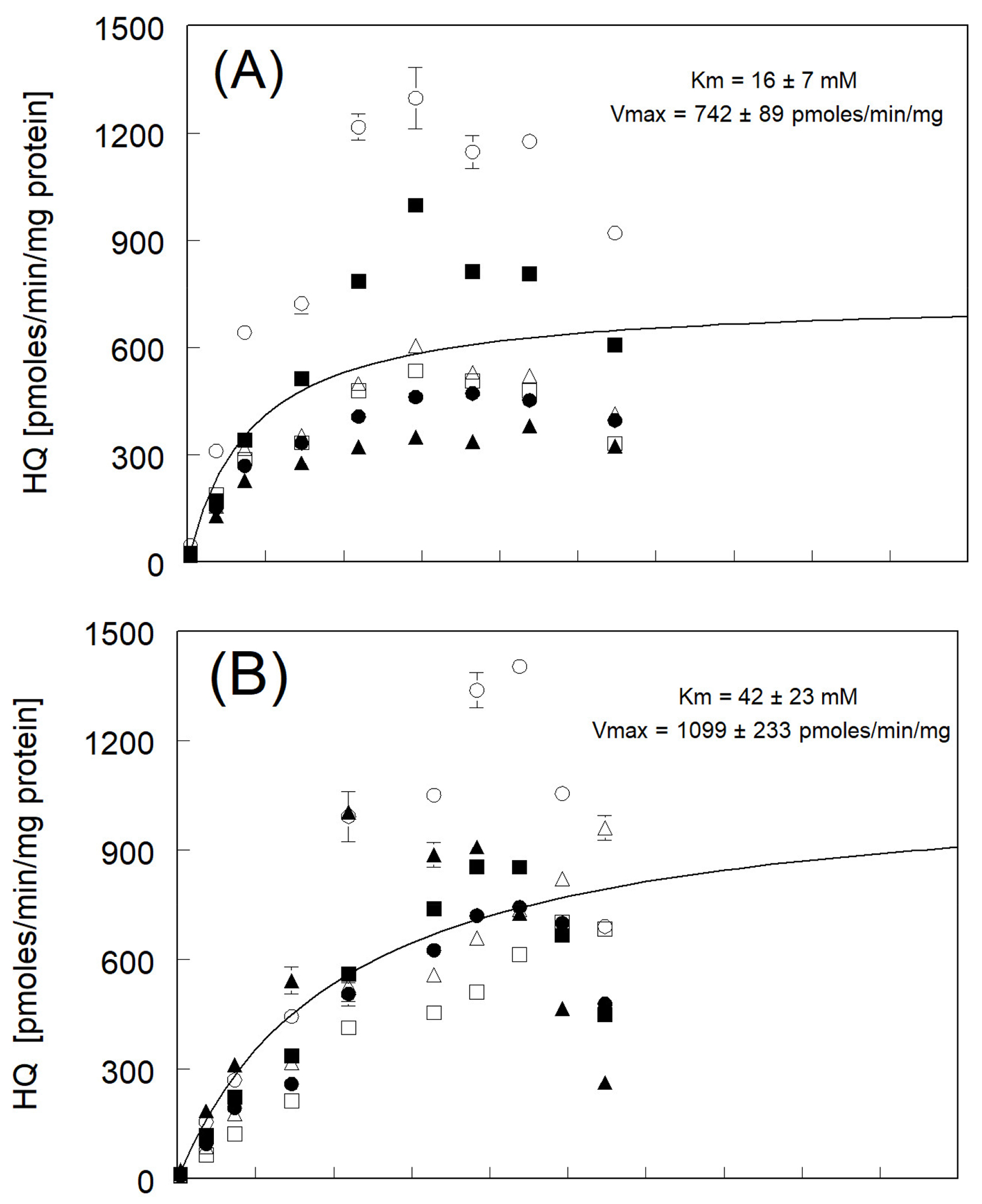

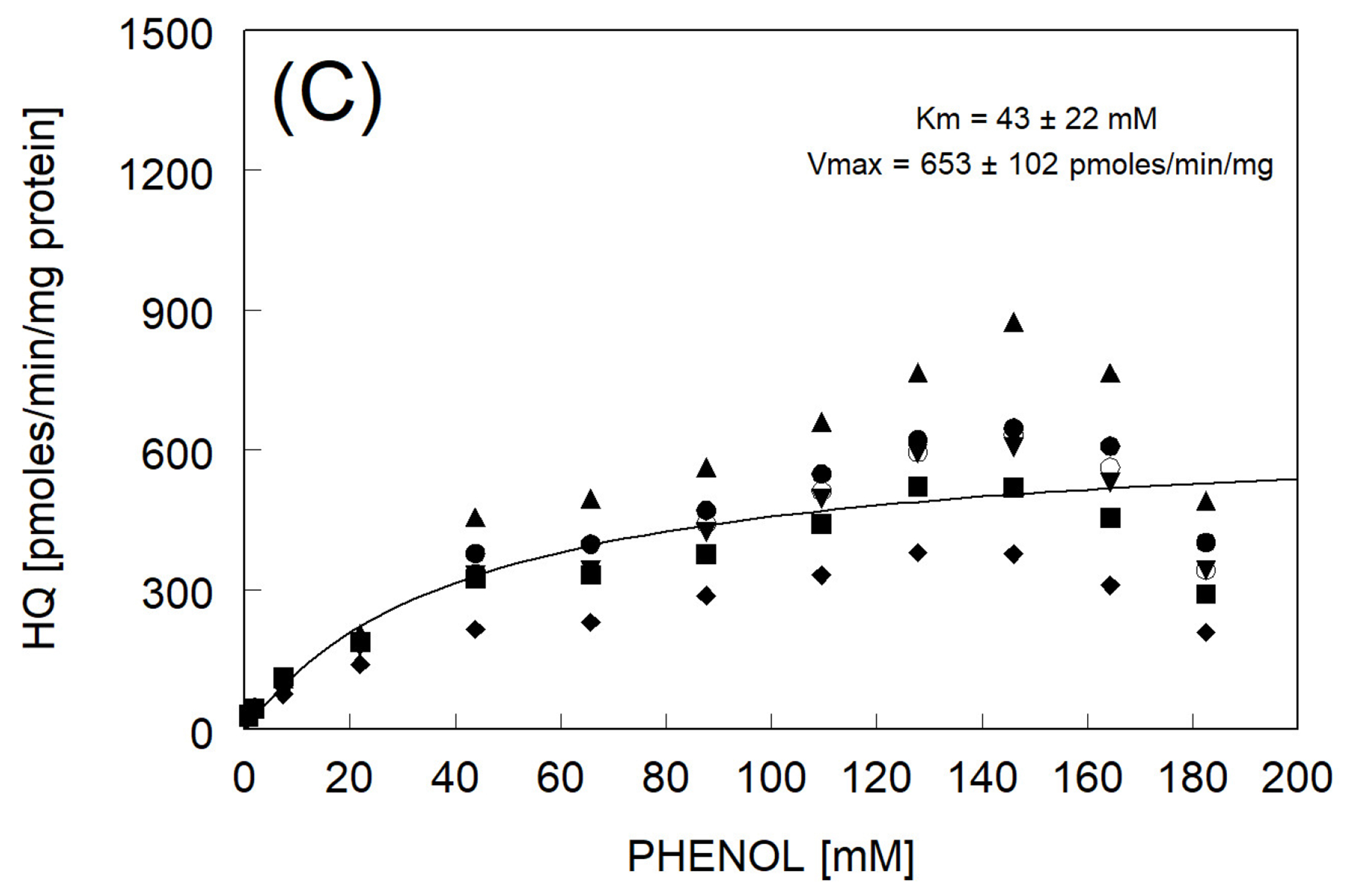
Rates of production of hydroquinone (HQ) resulting from the incubation of phenol with pre-spawn adult (**A**) RBT, (**B**) BKT, and (**C**) LKT microsomes at 11 °C. Each symbol represents the mean ± SE for triplicate microsome subsamples from each of six fish for each species. Open symbols represent female fish livers, and solid symbols represent male fish. The solid black line represents a fitted curve using average rate constants (over six fish) at each phenol concentration tested for calculation of the Michaelis–Menten constants Km and Vmax.

**Figure 2.**
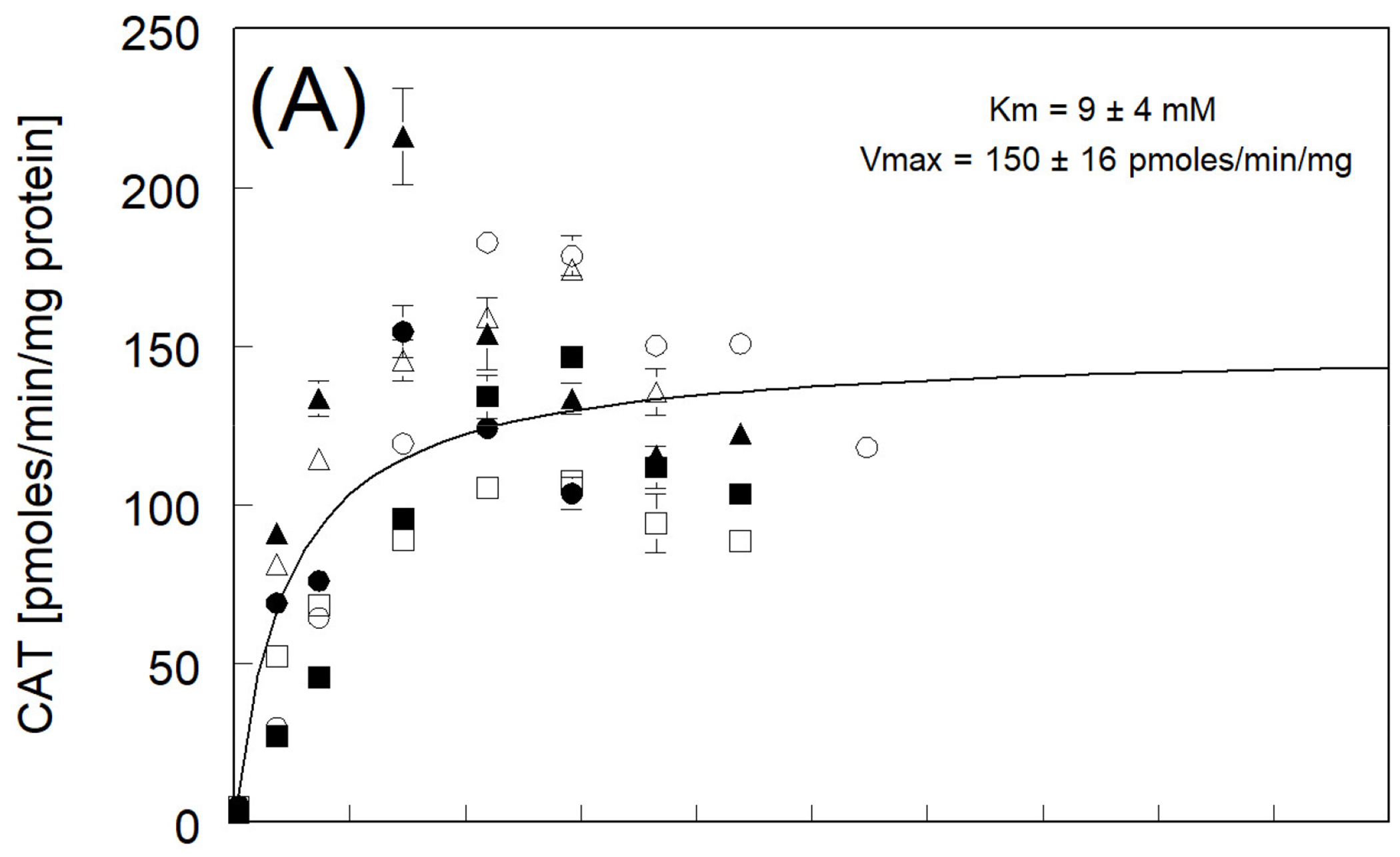

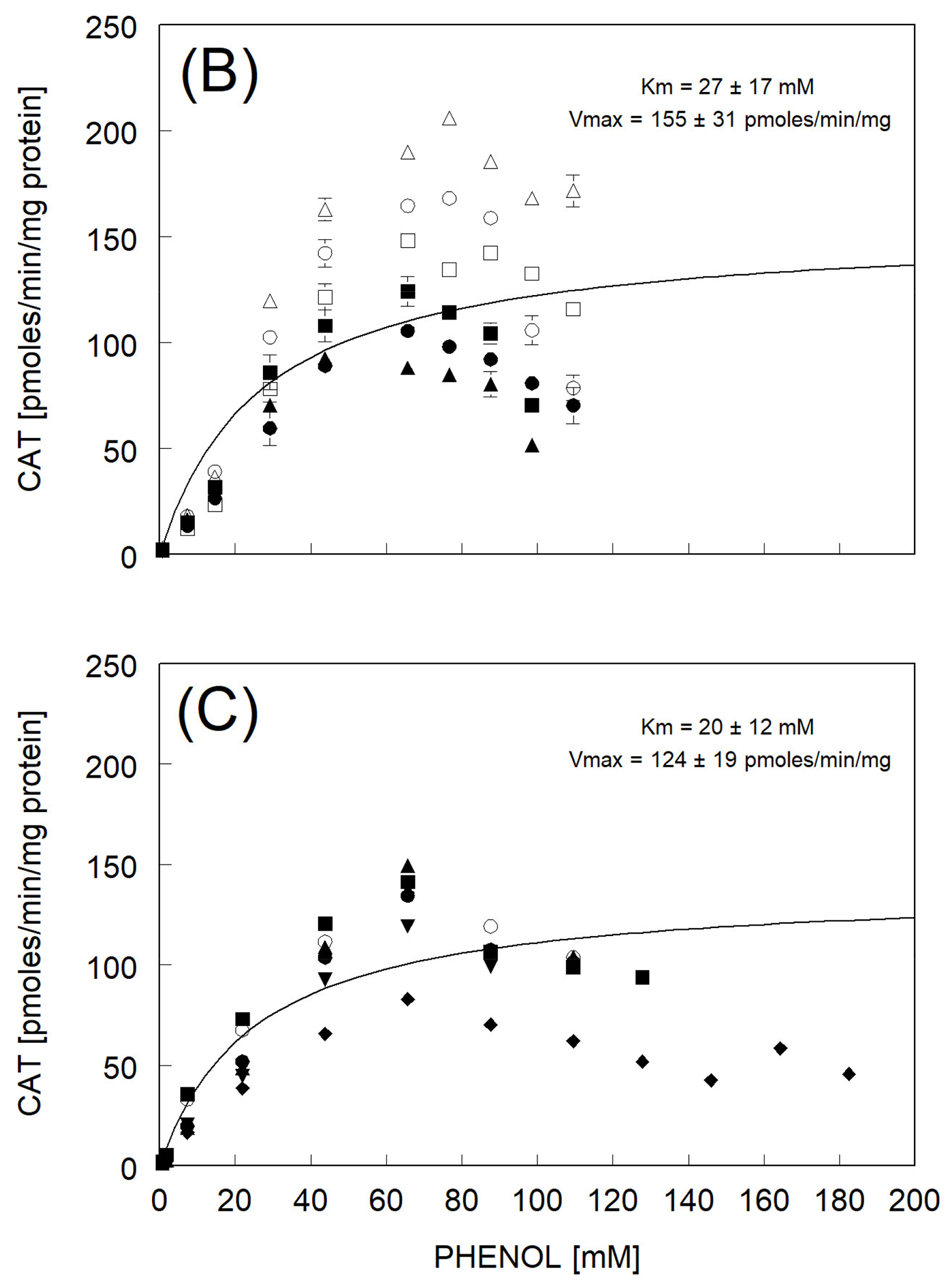
The rates of production of catechol (CAT) resulting from the incubation of phenol with pre-spawn adult (**A**) RBT, (**B**) BKT, and (**C**) LKT microsomes at 11 °C. Each symbol represents the mean ± SE for triplicate microsome subsamples from each of six fish for each species. Open symbols represent female fish livers, and solid symbols represent male fish. The solid black line represents a fitted curve using average rate constants (over six fish) at each phenol concentration tested for calculation of the Michaelis–Menten constants Km and Vmax.

**Figure 3.**
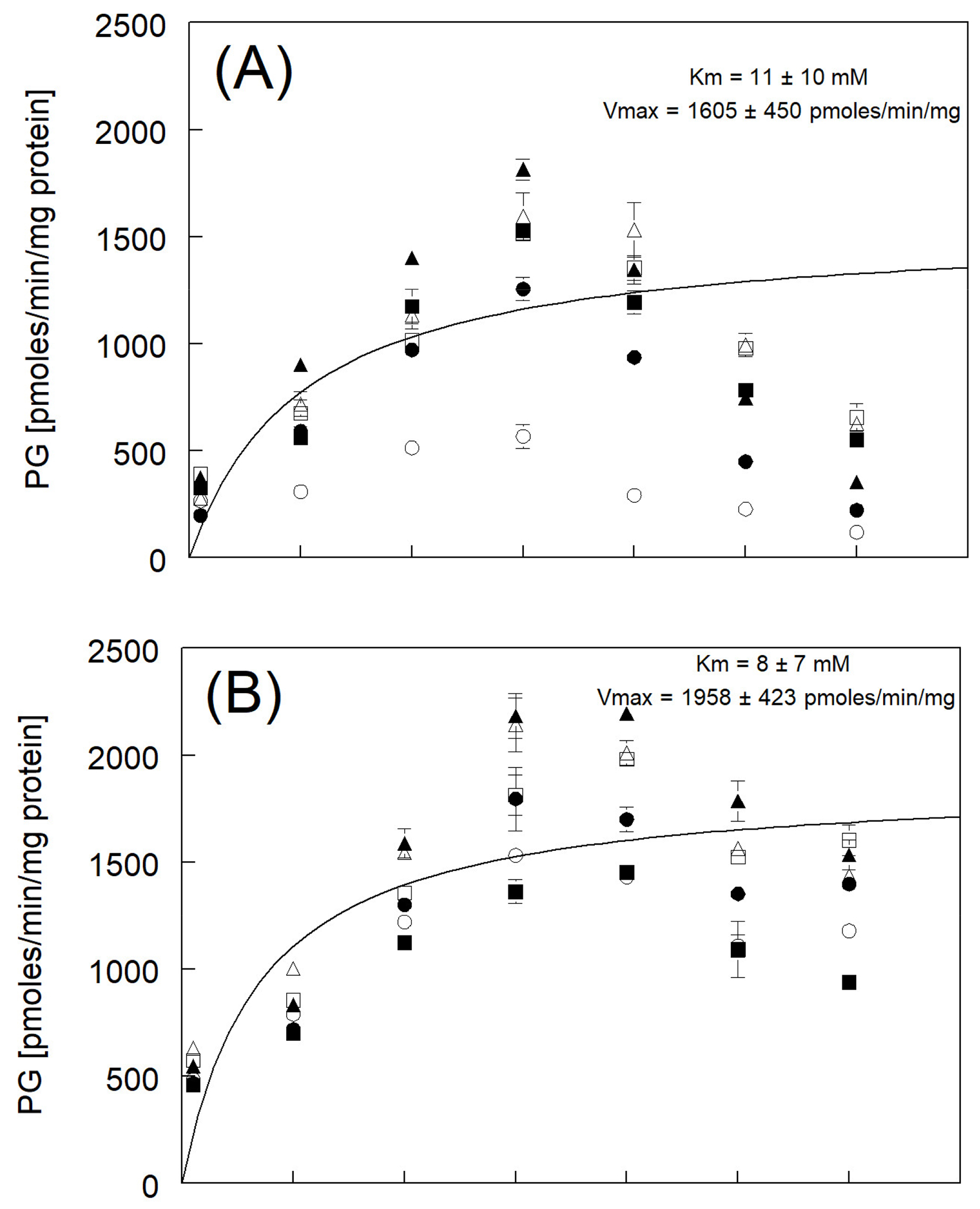

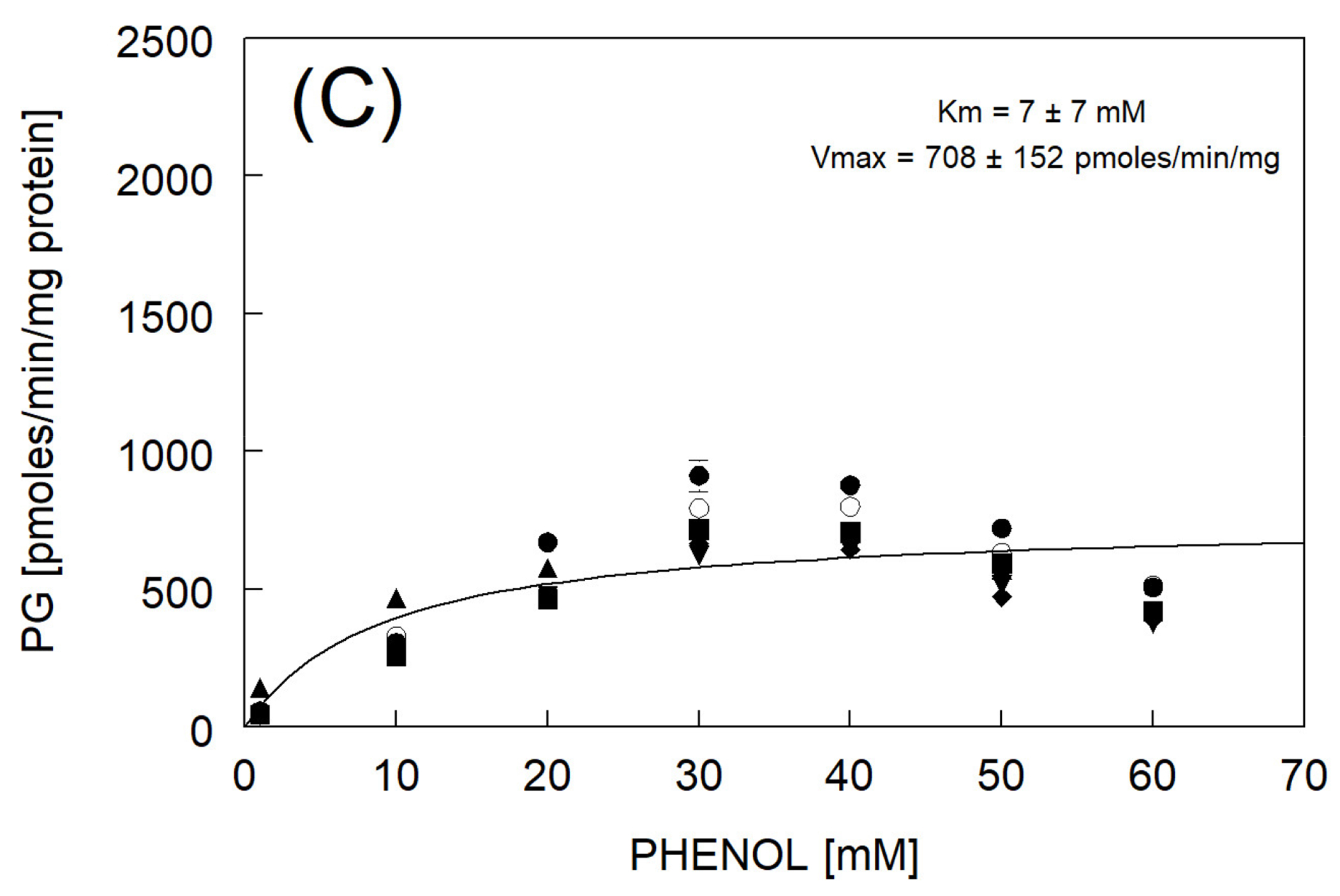
The rates of simultaneous production of phenylglucuronide (PG) resulting from the incubation of phenol with pre-spawn adult (**A**) RBT, (**B**) BKT, and (**C**) LKT microsomes at 11 °C. Each symbol represents the mean ± SE for triplicate microsome subsamples from each of six fish for each species. Open symbols represent female fish livers, and solid symbols represent male fish. The solid black line represents a fitted curve using average rate constants (over six fish) at each phenol concentration tested for calculation of the Michaelis–Menten constants Km and Vmax.

Liver microsomes prepared from RBT, BKT, and LKT were incubated with phenol at the physiologically relevant temperature of 11 °C. Nominal phenol concentrations (1–250 mM) were measured to be between 0.73 and 182.5 mM, consistent with previously observed reduction due to substrate/protein interaction at reduced temperature [44]. An apparent decrease in production of all three metabolites (HQ, CAT, and PG) occurred at the highest phenol concentrations for all three trout species (Figures 1–3). This may indicate denaturation of the enzyme at high substrate concentrations or some other substrate-mediated inhibitory effect.

For each of the three species of trout, there existed a relatively high degree of variability between fish in the rate of HQ and CAT formation. The variability was not attributable to gender differences for the pre-spawn adult fish tested; therefore, all fish were combined for the purpose of estimating the Km and Vmax for the production of metabolites.

### 3.2. Hydroquinone Formation

Parameter estimation (Km and Vmax) for the production of HQ from phenol for individual fish is shown in Table 2. Alternatively, the maximum production fitted to the average rates of HQ production across species was similar and ranged between 600 and 1100 pmoles HQ/min/mg protein (Figure 1). In RBT, linear production of HQ was observed up to 30 mM of phenol, followed by the apparent saturation reached at the maximum average near 750 pmoles HQ/min/mg protein (Figure 1A). Hydroquinone production with BKT microsomes resulted in the nearly linear production of HQ to 40 mM of phenol followed by apparent saturation, reaching a maximum average rate near 1100 pmoles/min/mg protein (Figure 1B). Production of HQ in LKT followed a similar pattern to that seen with BKT with nearly linear production demonstrated up to 45 mM of phenol and then followed by apparent saturation at a maximum average rate of only 653 pmoles HQ/min/mg protein (Figure 1C).

The variability in the maximum average rates of HQ production between individual fish across the three species ranged from a low of 300 to a high of 1200 pmoles/min/mg protein. It should be noted that the variability in the magnitude among the rates of fish hepatic microsomal metabolite production reported here is not uncommon between individual fish [44,45].

The measured apparent enzyme affinity (Km) across species for phenol conversion to HQ is greatest for RBT, with a lesser but similar affinity for BKT and LKT (Figure 1). No significant differences were determined in Vmax among the three species due to a high variability with a relative rank order of BKT > RBT = LKT.

### 3.3. Catechol Formation

The Michaelis–Menten kinetic constants for individual fish are shown in Table 3, allowing assessment of individual differences. Average rates, however, are similar to those in Figure 2. Catechol production in RBT followed a similar trend to that seen with HQ, but the average rates of production were lower with linearity to 20 mM of phenol and saturation to a maximum average rate of around 150 pmoles CAT/min/mg protein (Figure 2A). Production of CAT in the BKT was linear up to 40 mM of phenol, with saturation at a maximum average production rate of 150 pmoles CAT/min/mg protein, well below the average HQ formation rate (Figure 2B). The formation of CAT in LKT was again less than HQ and showed a linear increase to about 45 mM of phenol. It reached a maximum average production rate of only 124 pmoles CAT/min/mg protein at enzyme saturation, which was generally less than CAT production in RBT and BKT (Figure 2C). Again, as with HQ, no significant differences between trout species could be determined in the Vmax values for CAT production among the three species, with the relative rank order BKT = RBT > LKT. The Km values fitted to the average production rates of CAT showed RBT had the greatest apparent enzyme affinity, followed by the LKT and BKT (Figure 2).

### 3.4. Phenylglucuronide Formation

The production rates of PG in RBT reached apparent saturation at about 10 mM phenol, with a maximum average rate of approximately 1600 pmoles PG/min/mg protein (Figure 3A). One female trout had much lower apparent enzyme kinetics with a Vmax close to 440 and a high affinity Km of ~1 mM (Table 4, Figure 3A). Phenylglucuronide production in BKT also resulted in apparent saturation at 10 mM of phenol but with a maximum average rate of production close to 2000 pmoles/min/mg protein (Figure 3B). The rates of PG production for individual BKT microsome preparations ranged from 1450 to as high as 2200 pmoles/min/mg protein. Production of PG from phenol in LKT (Figure 3C) followed a similar pattern as other species with an apparent saturation around 10 mM of phenol but then reached a much lower maximum average rate near 750 pmoles/min/mg protein (ranging from 650 to 900 pmoles/min/mg protein).

The apparent Km and Vmax for the production of PG in individual RBT, BKT, and LKT microsomal preparations are shown in Table 4. The Km values fitted to the average production rates of PG from microsomal UDP-glucuronosyltransferase over a range of phenol concentrations (1–60 mM) indicated that the apparent enzyme affinity for the substrate was essentially the same across all three salmonids (Figure 3). The Vmax values fitted to the maximum average production rates of PG were significantly lower in the LKT than in RBT and BKT, which were similar in magnitude (Figure 3).

## 4. Discussion

Major hepatic microsomal Phase I and II biotransformation products of phenol (1–180 mM) in three species of pre-spawn adult salmonids at physiological temperature (11 °C) were quantified using sensitive analytical techniques able to resolve relatively low rates of production. Sample preparation and chromatographic conditions were optimized to achieve the chromatographic separation and sensitivity required for the analysis of these very labile products. Rate and capacity parameters (Km and Vmax) for the oxidation and conjugation of phenol in each of the trout species were calculated for future incorporation into PB-TK models for use in species extrapolation and environmental risk assessment [50].

The characterization of microsomes isolated from the livers of these three trout species showed that the microsomal protein on a per gram liver basis was significantly greater in RBT (*p* < 0.0001) than BKT and LKT, while no significant differences (*p* ≤ 0.05) were noted between the three species for P450 protein and EROD activity (Table 1). No sex-related differences were noted in any of the measurements. The higher microsomal protein levels in RBT may be indicative of a greater biotransformation capability through rate and capacity or through the number of different P450s present. However, more information is needed to explain the significance of these higher levels of microsomal protein in the RBT.

The microsomal protein, P450 protein, and EROD activity measured for pre-spawn adult RBT (Table 1) were similar to 18.5 ± 1.4 mg microsomal protein/g liver, 0.51 ± 0.04 nmoles/P450/mg microsomal protein, and 10.9 ± 12.3 pmoles ER/min/mg microsomal protein reported previously for juvenile (yearling) RBT by Kolanczyk and Schmieder [44]. This close agreement indicated that gender, age, and gonadal development seemed to have negligible effect on liver microsomal proteins. There appeared to be more variability in the results from pre-spawn adult RBT than in the juveniles, but this was likely due to the effect of pooling three or four individuals for each microsomal preparation in the juvenile fish experiments while keeping individuals separate in the current study.

### 4.1. Oxidative Metabolism (Phase I)

**Figure 4.**
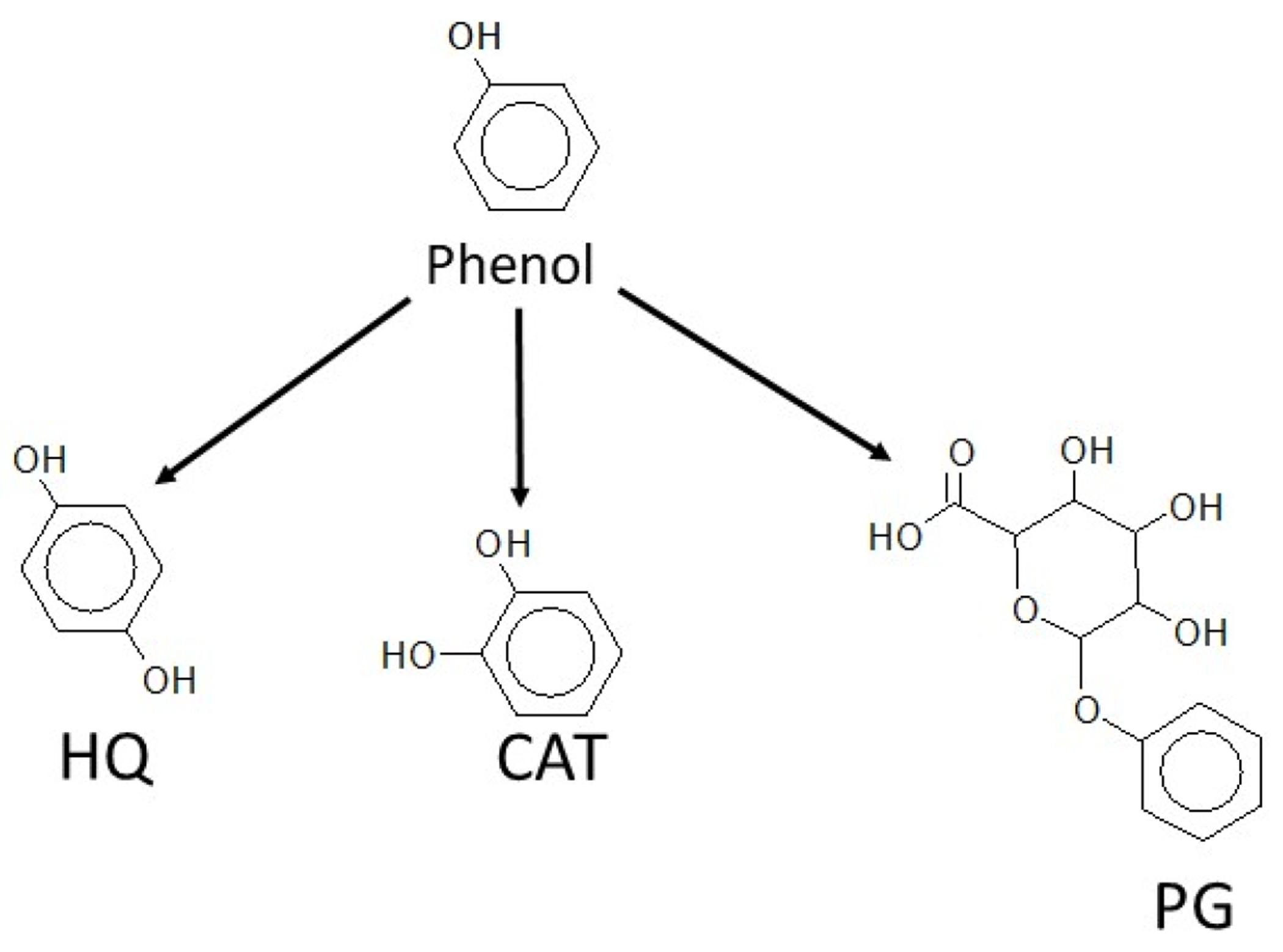
Metabolic conversion of phenol to hydroquinone (HQ), catechol (CAT), and phenylglucuronide (PG) in RBT, BKT, and LKT microsomes.

Hydroquinone was found to be the major Phase I metabolite produced in the hepatic biotransformation of phenol in all three species of trout (Figure 4). Catechol was also a significant Phase I metabolite resulting from phenol ring-hydroxylation. No other Phase I metabolites were found. The amount of CAT produced was typically five, seven, and five times less than the amount of HQ formed in RBT, BKT, and LKT microsomes, respectively. In comparison, the microsomal metabolism of phenol in rats resulted in product ratios (HQ:CAT) of 20:1 [51] and about 15:1 in both rats and mice [52]. The product ratios for the three trout species in this study ranged from 5:1 to 7:1 (HQ:CAT), suggesting there is greater relative ortho-hydroxylation in fish than in mammals.

Further oxidation of HQ to benzoquinone (BQ) results in a metabolite thought to be responsible for macromolecular binding in mammalian systems [53]. Benzoquinone was looked for via HPLC with electrochemical detection [44] but not detected in the present study, likely due to the presence of high concentrations of reducing equivalents in the microsomal system, as well as the high reactivity of BQ [54].

The kinetic rate constants, Km and Vmax, for HQ and CAT formation in all three trout species were calculated over a range of measured phenol concentrations (1–180 mM). There was a significant difference (*p* < 0.05) in Km between species for HQ formation, with the following rank order of apparent enzyme affinity for substrate (Figure 1): RBT (16 ± 7 mM) > BKT (42 ± 23 mM) = LKT (43 ± 22 mM). The RBT Km (9 ± 4 mM) for CAT formation was also different (*p* < 0.05) than BKT (27 ± 17 mM) and LKT (20 ± 12 mM) (Figure 2). All three species belong to the family Salmonidae (subfamily Salmoninae). RBT (*O. mykiss*) belongs to the genus *Oncorhynchus*, which includes 12 species of Pacific salmon and trout. Despite their names, BKT (*S. fontinalis*) and LKT (*S. namaycush*) belong to the genus *Salvelinus* and are known as char rather than trout. Differences in enzyme affinity (Km) appear to align with the genus. This observation is important, as PB-TK modelers often use the measured values for a surrogate species when the actual data are not available.

There was no significant difference in Vmax between species for HQ formation with RBT at 744 ± 154 pmoles HQ/min/mg protein, BKT at 1161 ± 165 pmoles HQ/min/mg protein, and LKT at 657 ± 81 pmoles HQ/min/mg protein (Figure 1), presumably due to the high degree of variation. Nonetheless, an apparent trend was observed whereby BKT had a higher Vmax than RBT or LKT. The Vmax for CAT formation was very similar between species: RBT = 163 ± 12 pmoles CAT/min/mg protein, BKT = 167 ± 26 pmoles CAT/min/mg protein, and LKT = 134 ± 7 pmoles CAT/min/mg protein (Figure 2). The enzyme capacity (Vmax) for the ring hydroxylation of phenol to HQ and CAT is conserved across the three species. Each species could presumably serve as a surrogate for one another with respect to this reaction.

The Michaelis–Menten kinetic rate constants obtained in this study for pre-spawn adult RBT were compared to those previously determined for juvenile RBT. Average Km values measured in pre-spawn adult RBT for the formation of HQ (15 ± 2 mM) and CAT (12 ± 3 mM) were not significantly different from those previously determined in juvenile RBT for the formation of HQ (14 ± 1 mM) and CAT (10 ± 1 mM) [44]. There was also no difference in the average Vmax values obtained for HQ and CAT formation in pre-spawn adult rainbow trout (744 ± 154 pmoles HQ/min/mg protein and 163 ± 12 pmoles CAT/min/mg protein) and juvenile RBT (552 ± 71 pmoles HQ/min/mg protein and 161 ± 15 pmoles CAT/min/mg protein) reported by Kolanczyk and Schmieder [44]. The microsomal preparations from the juvenile fish originated from fish weighing 165 ± 10 g, a HSI of 1.3 ± 0.1%, and 18.5 ± 0.6 mg microsomal protein/g liver [44] as compared to the RBT of the current study, weighing 754 ± 72 g, a HSI of 1.4 ± 0.5%, and 17.0 ± 0.9 mg microsomal protein/g liver. Therefore, the developmental size and age of RBT did not appear to influence liver microsomal biotransformation rates. While the comparison between juvenile and pre-spawn adult RBT agrees favorably, the impact of sexual maturation on xenobiotic metabolism in adult fish needs further investigation.

Rainbow trout enzymes had a greater affinity for phenol than BKT or LKT, which resulted in RBT having a higher rate of formation for HQ and CAT at the lower concentrations of phenol. All three trout species produced HQ and CAT at similar rates at high phenol concentrations (at saturation) with similar Vmax across the three trout species. This could potentially result in RBT being more susceptible to low concentrations of phenol due to the formation of potentially toxic metabolites (HQ and CAT). The 4-day LC_50_ for RBT was reported as 10.5 mg/L phenol [55] and 0.097 mg/L HQ [56], resulting in a 100-fold increase in toxicity as mediated through the biotransformation of phenol. Yet, at higher phenol concentrations (above 30 mM), all three trout species may show similar susceptibility, as they produce equal amounts of HQ and CAT.

### 4.2. Deactivation (Phase II)

Earlier studies with phenolic compounds have shown that PG is a major conjugative metabolite produced by hepatic microsomes in fish [22]. Sulfate conjugates of phenol are also present in fish [39] but in very small amounts [43]. In the present study, PG was identified in the microsomal preparations and was the only primary Phase II metabolite measured in the three trout tested (Figure 4). The maximum average rates of production for PG over a range of concentrations of phenol (1–60 mM) in the three trout ranged from 2 to 12 times higher than the rates for HQ and CAT production, which emphasizes its importance in deactivation of the very labile HQ and CAT. There were no significant statistical differences (*p* ≤ 0.05) in Km values between species for PG formation and, therefore, no differences in enzyme affinity, but the affinity for all three species, based on the low Km value, was quite high.

There was a significant (*p* ≤ 0.05) difference in Vmax between species for PG formation, with RBT (1605 ± 450 pmoles PG/min/mg protein) and BKT (1958 ± 423 pmoles PG/min/mg protein) essentially the same while LKT (708 ± 152 pmoles PG/min/mg protein) was significantly lower (Figure 3).

The Km values for the conjugation of phenol to PG in RBT, BKT, and LKT indicate that the hepatic P450s of these species have a similar affinity for PG. However, the significantly lower Vmax for PG conjugation in LKT could result in saturation of their conjugation system at lower concentrations of phenol. Therefore, LKT may be more susceptible to the toxic effects of Phase I metabolites (HQ and CAT) formed from high concentrations of phenol that exceed their Vmax, while RBT and BKT can tolerate higher phenol concentrations before saturating the PG conjugation system.

## 5. Conclusions

This study was done to gain an understanding of the differences in biotransformation rates between fish and their importance in toxicology and bioaccumulation for use in species extrapolation. The cold water species tested in this study, RBT, BKT, and LKT, all metabolized phenol through the same pathway, resulting in formation of the oxidative metabolites HQ and CAT and the conjugative metabolite PG. The metabolism of phenol over a range of concentrations was quantified, and the Michaelis–Menten kinetics constants, Km and Vmax, for the formation of HQ, CAT, and PG were calculated. For the production of HQ and CAT, an increased affinity of the substrate (Km) was found in RBT over both BKT and LKT; however, there was no observed differences in the capacity (Vmax) to produce HQ and CAT across the three species. There was no observed Km difference across species to produce PG from phenol; however, the Vmax for LKT was significantly lower than that of RBT and BKT. Despite the similarity of the pathway, there are important differences in the rate and metabolic capacities that should be considered when generalizing across species. Comparative studies characterizing metabolic pathways and rates across fish species are needed as the foundation for the development of predictive models. A better understanding of the rate and capacity trends across species for generalized enzymatic processes such as ring-hydroxylation and glucuronidation is required to validate where values may be extrapolated across species. This research effort provides a step in the right direction. Comparative analysis for additional substrates that undergo ring-hydroxylation and glucuronidation would be needed to support the observed similarity and difference trends as observed for the species in this study. In the future, an extensive database of these rate and capacity parameters on important fish species for selected chemical structures will be needed to allow the effective use of predictive models for reactive, biotransformation chemicals in aquatic toxicology and environmental risk assessment.

## Supplementary Material

Supplement1

## Figures and Tables

**Table 1.** A summary of fish size, sex, liver weight, and hepatosomatic indices (HSI) for six fish of each species used for the preparation of hepatic microsomes. Triplicate samples of each microsomal preparation were characterized for the microsomal protein content, P450 protein concentration, and EROD activity.

	Species	Sex	Fish Weight (g)	Liver Weight (g)	HSI (%)	Microsomal Protein (mg/g Liver)	P450 Protein (nmol/mg Microsomal Protein)	EROD (pmol/min/mg Microsomal Protein)
fish #1	Rainbow	male	913	10.3	1.1	16.5	0.50 **	18.2
fish #2	Rainbow	male	863	9.1	1.1	12.7	0.52 **	32.7
fish #3	Rainbow	male	502	5.6	1.1	17.5	0.54 **	2.9
fish #4	Rainbow	female	861	18.9	2.2	18.0	0.17 **	2.8
fish #5	Rainbow	female	823	9.9	1.2	18.3	0.35 **	7.6
fish #6	Rainbow	female	561	10.2	1.8	19.2	0.22 **	1.4

avg ± std err			754 ± 72	10.7 ± 1.8	1.4 ± 0.5	17.0 ± 0.9 *	0.38 ± 0.07	10.9 ± 5.0

fish #1	Brook	male	1376	19.3	1.4	8.4	0.57 ***	6.8
fish #2	Brook	male	1093	14.2	1.3	7.9	0.58 ***	5.2
fish #3	Brook	male	987	11.7	1.2	11.0	0.61 ***	9.6
fish #4	Brook	female	1282	29.2	2.3	8.0	0.47 ***	3.0
fish #5	Brook	female	974	18.4	1.9	9.8	0.38 ***	2.4
fish #6	Brook	female	779	12.8	1.6	9.5	0.47 ***	6.3

avg ± std err			1082 ± 89	17.6 ± 2.6	1.6 ± 0.4	9.1 ± 0.5 *	0.51 ± 0.04	5.6 ± 1.1

fish #1	Lake	male	954	10.3	1.1	11.1	0.49	11.3
fish #2	Lake	male	1140	15.0	1.3	13.3	0.41	5.7
fish #3	Lake	male	1209	13.9	1.1	11.1	0.40	5.3
fish #4	Lake	male	1227	20.9	1.7	8.0	0.38	8.3
fish #5	Lake	male	1592	18.8	1.2	12.0	0.48	3.8
fish #6	Lake	female	893	10.4	1.2	9.4	0.58	6.6

avg ± std err			1169 ± 101	14.9 ± 1.8	1.3 ± 0.2	10.8 ± 0.8 *	0.46 ± 0.03	6.8 ± 1.1

* Microsomal protein mg/g liver (*p* < 0.0001) RBT different from BKT and LKT.

** P450 (*p* = 0.0091) RBT males (0.52 ± 0.01 nmoles/mg protein) different from RBT females (0.25 ± 0.06 nmoles/mg protein).

*** P450 (*p* = 0.0138) BKT males (0.58 ± 0.01 nmoles/mg protein) different from BKT females (0.44 ± 0.03 nmoles/mg protein).

**Table 2.** The Michaelis–Menten kinetic constants for phenol ring-hydroxylation to hydroquinone in pre-spawn adult trout calculated over a range of phenol concentrations. Constants are presented based on individual fish, as an average of the individual fish, and fitted to the average rate at each phenol concentration for all six fish. Km (mM) and Vmax (pmoles/min/mg microsomal protein).

RAINBOW TROUT		HYDROQUINONE	
	SEX	Km	Vmax
fish #1	male	15 ± 4	525 ± 35
fish #2	male	22 ± 14	1013 ± 193
fish #3	male	13 ± 3	405 ± 20
fish #4	female	17 ± 9	1383 ± 191
fish #5	female	11 ± 6	527 ± 64
fish #6	female	15 ± 7	608 ± 72

avg of individual Km and Vmax ± std err (N = 6)		15 ± 2 **	744 ± 154
Km and Vmax fitted to avg rate		16 ± 7	742 ± 89
BROOK TROUT		HYDROQUINONE	
	SEX	Km	Vmax

fish #1	male	54 ± 34	1038 ± 283
fish #2	male	38 ± 29	997 ± 278
fish #3	male	18 ± 16	956 ± 234
fish #4	female	45 ± 39	1654 ± 574
fish #5	female	243 ± 112	2277 ± 782
fish #6	female	232 ± 106	2800 ± 942

avg of individual Km and Vmax ± std err (N = 6)		105 ± 42	1620 ± 316
avg of individual Km and Vmax ± std err (N = 4) *		38 ± 8 **	1161 ± 165
Km and Vmax fitted to avg rate		42 ± 23	1099 ± 233
LAKE TROUT		HYDROQUINONE	
	SEX	Km	Vmax

fish #1	male	46 ± 21	680 ± 119
fish #2	male	30 ± 15	526 ± 68
fish #3	male	55 ± 29	966 ± 176
fish #4	male	45 ± 24	656 ± 109
fish #5	male	31 ± 16	381 ± 53
fish #6	female	46 ± 25	680 ± 119

avg of individual Km and Vmax ± std err (N = 6)		42 ± 4 **	657 ± 81
Km & Vmax fitted to avg rate		43 ± 22	653 ± 102

* Average of rates based on fish 1–4 only, with large almost linear constants for fish 5 and 6.

** The Km HQ (*p* < 0.01) RBT was different from BKT and LKT.

**Table 3.** The Michaelis–Menten kinetic constants for phenol ring-hydroxylation to catechol in prespawn adult trout calculated over a range of phenol concentrations. Constants are presented based on individual fish, as an average of the individual fish, and fitted to the average rate at each phenol concentration for all six fish. Km (mM) and Vmax (pmoles/min/mg microsomal protein).

RAINBOW TROUT		CATECHOL	
	SEX	Km	Vmax
fish #1	male	8 ± 6	146 ± 32
fish #2	male	24 ± 16	166 ± 38
fish #3	male	6 ± 6	187 ± 39
fish #4	female	18 ± 12	190 ± 35
fish #5	female	8 ± 3	111 ± 7
fish #6	female	8 ± 3	180 ± 15

avg of individual Km and Vmax ± std err N = 6)		12 ± 3 **	163 ± 12
Km and Vmax fitted to avg rate		9 ± 4	150 ± 16
BROOK TROUT		CATECHOL	
	SEX	Km	Vmax

fish #1	male	28 ± 16	118 ± 22
fish #2	male	25 ± 18	137 ± 32
fish #3	male	17 ± 13	97 ± 19
fish #4	female	23 ± 20	171 ± 43
fish #5	female	45 ± 25	205 ± 45
fish #6	female	42 ± 20	273 ± 51

avg of individual Km and Vmax ± std err (N = 6)		30 ± 5	167 ± 26
avg of individual Km and Vmax ± std err (N = 4) *		23 ± 2 **	131 ± 16
Km and Vmax fitted to avg rate		27 ± 17	155 ± 31
LAKE TROUT		CATECHOL	
	SEX	Km	Vmax

fish #1	male	31 ± 18	154 ± 32
fish #2	male	16 ± 10	140 ± 21
fish #3	male	25 ± 18	143 ± 30
fish #4	male	32 ± 18	142 ± 28
fish #5	male	36 ± 15	113 ± 20
fish #6	female	10 ± 7	112 ± 13

avg of individual Km and Vmax ± std err (N = 6)		25 ± 4 **	134 ± 7
Km and Vmax fitted to avg rate		20 ± 12	124 ± 19

* Average of rates based on fish 1–4 only.

** Km CAT (*p* < 0.05) RBT different from BKT and LKT.

**Table 4.** The Michaelis–Menten kinetic constants for the glucuronidation of phenol to phenylglucuronide in pre-spawn adult trout calculated over a range of phenol concentrations. Constants are presented based on individual fish, as an average of the individual fish, and fitted to the average rate at each phenol concentration for all six fish. Km (mM) and Vmax (pmoles/min/mg microsomal protein).

RAINBOW TROUT		PHENYLGLUCURONIDE	
	SEX	Km	Vmax
fish #1	male	10 ± 9	1383 ± 369
fish #2	male	15 ± 16	1935 ± 773
fish #3	male	7 ± 7	1872 ± 446
fish #4	female	1 ± 1	438 ± 80
fish #5	female	17 ± 15	2076 ± 742
fish #6	female	8 ± 8	1591 ± 399

avg of individual Km and Vmax ± std err (N = 6)		10 ± 3	1549 ± 245 *
Km and Vmax fitted to avg rate		11 ± 10	1605 ± 450
BROOK TROUT		PHENYLGLUCURONIDE	
	SEX	Km	Vmax

fish #1	male	7 ± 6	1771 ± 315
fish #2	male	5 ± 5	1431 ± 250
fish #3	male	8 ± 7	2204 ± 449
fish #4	female	9 ± 7	1811 ± 425
fish #5	female	7 ± 6	1968 ± 322
fish #6	female	6 ± 6	2108 ± 416

avg of individual Km and Vmax ± std err (N = 6)		7 ± 1	1882 ± 113 *
Km and Vmax fitted to avg rate		8 ± 7	1958 ± 423
LAKE TROUT		PHENYLGLUCURONIDE	
	SEX	Km	Vmax

fish #1	male	9 ± 10	887 ± 236
fish #2	male	10 ± 11	727 ± 193
fish #3	male	5 ± 2	726 ± 70
fish #4	male	8 ± 8	627 ± 142
fish #5	male	11 ± 9	738 ± 173
fish #6	female	11 ± 10	815 ± 201

avg of individual Km and Vmax ± std err (N = 6)		9 ± 1	753 ± 36 *
Km and Vmax fitted to avg rate		7 ± 7	708 ± 152

* Vmax PG (*p* < 0.01) LKT different from RBT and BKT.

## Data Availability

All data from the present study are publicly available at the US Environmental Protection Agency Environmental Dataset Gateway (https://edg.epa.gov/metadata/catalog/main/home.page).
